# Combined Pre- and Posttreatment of Paraoxon Exposure

**DOI:** 10.3390/molecules25071521

**Published:** 2020-03-27

**Authors:** Dietrich E Lorke, Syed M Nurulain, Mohamed Y Hasan, Kamil Kuča, Georg A Petroianu

**Affiliations:** 1 Department of Anatomy and Cellular Biology, College of Medicine and Health Sciences, Khalifa University, P O Box 127788, Abu Dhabi, UAE; 2 Herbert Wertheim College of Medicine, Department of Cellular Biology & Pharmacology, Florida International University, University Park GL 495, 11200 SW 8th St, Miami, FL 33199, USA; georg.petroianu@ku.ac.ae; 3 Bio Science Department, COMSATS Institute of Information Technology, Bio Sciences Block, CUI, Park Road, Tarlai Kalan, Islamabad 45550, Pakistan; syed.nurulain@comsats.edu.pk; 4 Department of Pharmacology & Therapeutics, College of Medicine and Health Sciences, UAE University, Al Ain 15551, UAE; my.baniyas@moe.gov.ae; 5 Department of Chemistry, Faculty of Science, University of Hradec Kralove, Rokitanského 62/26, 500 03 Hradec Kralove, Czech Republic; kamil.kuca@uhk.cz; 6 Department of Pharmacology, College of Medicine and Health Sciences, Khalifa University, P O Box 127788, Abu Dhabi, UAE

**Keywords:** carbamates, cholinesterase, cox analysis, paraoxon, oximes, organophosphate, pretreatment, prophylaxis, rat

## Abstract

Aims: Organophosphates (OPCs), useful agents as pesticides, also represent a serious health hazard. Standard therapy with atropine and established oxime-type enzyme reactivators is unsatisfactory. Experimental data indicate that superior therapeutic results can be obtained when reversible cholinesterase inhibitors are administered before OPC exposure. Comparing the protective efficacy of five such cholinesterase inhibitors (physostigmine, pyridostigmine, ranitidine, tacrine, or K-27), we observed best protection for the experimental oxime K-27. The present study was undertaken in order to determine if additional administration of K-27 immediately after OPC (paraoxon) exposure can improve the outcome. Methods: Therapeutic efficacy was assessed in rats by determining the relative risk of death (RR) by Cox survival analysis over a period of 48 h. Animals that received only pretreatment and paraoxon were compared with those that had received pretreatment and paraoxon followed by K-27 immediately after paraoxon exposure. Results: Best protection from paraoxon-induced mortality was observed after pretreatment with physostigmine (RR = 0.30) and K-27 (RR = 0.34). Both substances were significantly more efficacious than tacrine (RR = 0.67), ranitidine (RR = 0.72), and pyridostigmine (RR = 0.76), which were less efficacious but still significantly reduced the RR compared to the no-treatment group (paraoxon only). Additional administration of K-27 immediately after paraoxon exposure (posttreatment) did not further reduce mortality. Statistical analysis between pretreatment before paraoxon exposure alone and pretreatment plus K-27 posttreatment did not show any significant difference for any of the pretreatment regimens. Conclusions: Best outcome is achieved if physostigmine or K-27 are administered prophylactically before exposure to sublethal paraoxon dosages. Therapeutic outcome is not further improved by additional oxime therapy immediately thereafter.

## 1. Introduction

Poisonings with organophosphorus compounds (OPCs) are amongst the most frequent intoxications worldwide, a fact that is related to their extensive use for diverse purposes and their easy availability (see [1] for review). Whereas many of these compounds (hydrolic fluids, lubricants, or plasticisers) do not inhibit cholinesterases and are therefore of little acute toxicological concern, insecticides and acaricides are highly toxic. It is estimated that approximately 200,000 people die every year due to OPC pesticide intoxications [2,3]. There are also numerous examples of OPCs being misused in criminal poisonings, terrorist attacks, and chemical warfare [4,5]. The OPC nerve agents sarin and tabun were employed against Iranian troops and civilians during the 1980–1988 Iraq/Iran war, resulting in hundreds of fatalities [6,7,8]. Three terrorist attacks with the nerve agents sarin and venomous agent X (VX) in the Japanese cities of Matsumoto, Osaka, and Tokyo killed over 20 people and caused a high number of casualties [4,9]. An example of a chemical weapon that is relatively easy to create is the improvised bomb produced by Hamas containing pesticides [10,11]. Reports of suspected and confirmed gas attacks in the Syrian Civil War [12,13,14] and allegations that the terrorist group ISIS may have stolen and employed sarin in Libya [15] document the ongoing serious threat not only to civilians but also to rescue personnel.

The acute toxicity of OPCs is due to their ability to phosphylate (i.e., phosphorylate or phosphonylate) a serine residue at the active site of the enzyme acetylcholinesterase (AChE), thereby rendering it inactive. AChE is the enzyme responsible for terminating the synaptic action of the neurotransmitter acetylcholine (ACh). As a consequence of this inhibition, ACh accumulates at cholinergic synapses and stimulates muscarinic and nicotinic receptors, thereby causing a cholinergic crisis. Muscarinic signs and symptoms can be memorized by the mnemonic DUMBBELLS (diarrhea, urination, miosis, bronchorrhea, bronchospasms, emesis, lacrimation, laxation, and sweating); nicotinic features consist of tachycardia, muscle fasciculations, and cramps, as well as paralysis; and central nervous system symptoms comprising dizziness, seizures, and coma. Patients generally die due to respiratory or multi-organ failure, cardiovascular collapse, or generalized seizures [16,17,18]; for review, see [6,19,20].

Phosphylated AChE can, however, be reactivated by oximes, which are nucleophilic agents removing the phosphyl moiety from the AChE molecule [21]. Therefore, the standard therapy of OPC intoxications consists of an oxime restoring the enzymatic function of the AChE enzyme in combination with atropine blocking muscarinic receptor stimulation and a benzodiazepine controlling convulsions [19,22,23]. However, the efficacy of this postexposure treatment, especially in the case of pesticide exposure, is still a matter of debate [15,24], and AChE can only be reactivated within a limited time window, due to a rapid dealkylation process called aging [5,25,26].

In situations where prophylaxis is feasible, better therapeutic results are achieved by pretreatment with reversible AChE inhibitors. The rationale of this approach is that these inhibitors, generally carbamates, reversibly bind to the active site of the AChE molecule, thereby protecting it from irreversible phosphylation by OPCs (reviewed by [26]). This strategy was already conceived in the 1940s by Koster (1946), and its efficacy has been demonstrated by numerous in vitro and in vivo experiments (reviewed by [26]). The most efficacious pretreatment agent is the carbamate physostigmine, the use of which is, however, hampered by its passage into the brain, resulting in serious behavioral side effects (reviewed by [5]), precluding its application in situations requiring critical decision making. The carbamate pyridostigmine, which does not cross the blood brain barrier, has therefore been distributed to soldiers and civilians during the 1991 Gulf War to protect them from nerve gas attacks [27,28,29]. Afterwards, the United States Food and Drug Administration (FDA) approved the oral administration of pyridostigmine when soman exposure is anticipated [30]. However, prophylactic application of pyridostigmine is only efficacious if it is followed by atropine plus oxime treatment after exposure to nerve gases and is hampered by numerous side effects (reviewed by [5]), which has encouraged research into superior alternatives protecting from OPC intoxication.

**Figure 1:**
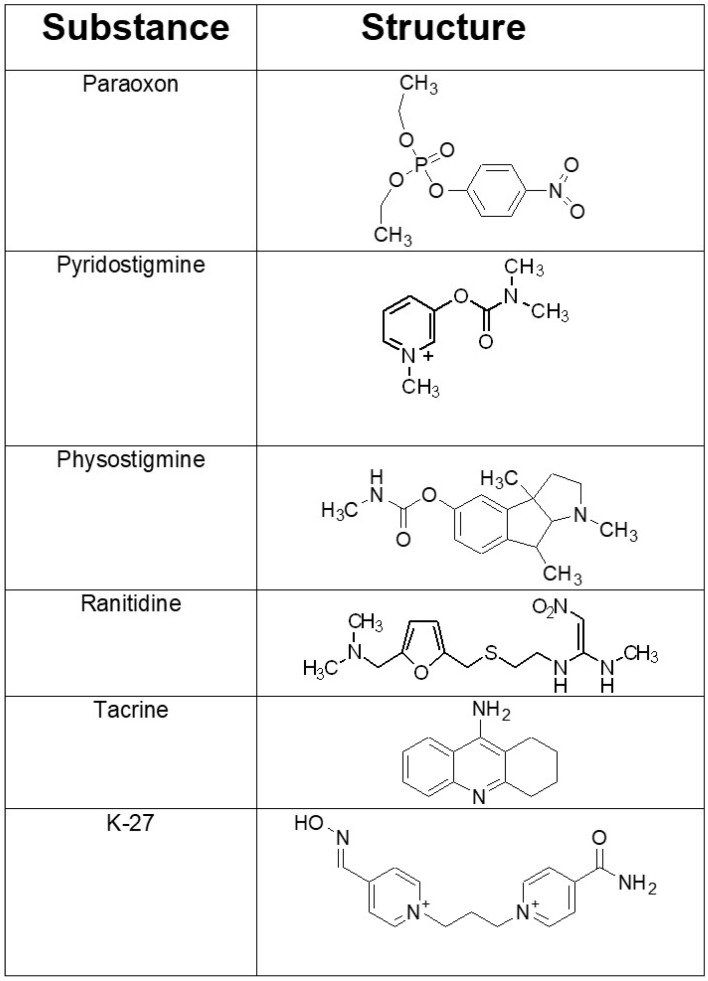
Chemical structures of the organophosphorus compound paraoxon employed and of the investigated acetylcholinesterase (AChE) inhibitors that were administered prophylactically before paraoxon exposure. Pyridostigmine is a potent cholinesterase inhibitor, which does not cross the blood-brain barrier. It is the only FDA-approved compound for prophylaxis prior to soman exposure. Physostigmine and tacrine are AChE inhibitors that enter the central nervous system. They have been used to improve the cognitive performance in Alzheimer’s disease. The histamine type 2 (H2) receptor blocker ranitidine is used clinically to inhibit gastric acid secretion. K-27 belongs to a group of newly developed oxime-type AChE reactivators with promising in vitro and in vivo characteristics.

During the last few years we have assessed a number of cholinesterase inhibitors (Figure 1) which are either already utilized therapeutically for other indications (physostigmine, ranitidine, tiapride, tacrine, amiloride, metoclopramide, and methylene blue) or which have been synthesized as future medications (7-methoxytacrine, K-27). When administered before the OPCs ethyl-paraoxon [31], methyl-paraoxon [32], DFP [33], terbufos [34], azinphos-methyl [35], and dicrotophos [36], best reduction in mortality was achieved by physostigmine and K-27, compounds that were significantly more efficacious than the FDA-approved compound pyridostigmine [26].

K-27 (Figure 1), a bisquaternary asymmetric pyridinium aldoxime containing only one functional aldoxime group in position four of the pyridine ring [37], belongs to a group of recently synthesized and tested oximes (K-oximes) [38,39,40,41,42,43], which significantly protect from OPC-induced mortality when given after exposure to a wide range of chemically diverse OPCs [42,44,45,46], reviewed by [47,48]. Comparable to other oximes, K-oximes not only reactivate phosphylated AChE but also themselves inhibit AChE activity [47,49].

It is generally assumed that the therapeutic outcome of a pretreatment regimen is improved by subsequent posttreatment after OPC exposure [50,51,52,53,54]. The present experiment has therefore been undertaken in order to test if application of the experimental oxime K-27 immediately after exposure to sublethal dosages of the OPC paraoxon improves the mortality of animals pretreated with a group of reversible OPC inhibitors (physostigmine, pyridostigmine, ranitidine, tacrine, or K-27). For comparison of efficacies, Cox survival analysis has been chosen, which is based on the relative risk of death (RR). This analysis is a general regression model, which assumes that the underlying hazard rate is a function of independent variables. The Cox regression model allows for a statistical analysis of different survival curves. Instead of only looking at the survival at one predetermined time point, the comparison of two survival curves measured over an extended period provides additional information. Moreover, the Cox proportional hazards model also allows for the analysis of several factors of known or likely importance for the survival of the animals. In our case, these covariates have been paraoxon dose and type of pretreatment.

## 2. Results

### 2.1. Mortalities

Survival of the experimental animals depended both upon the substance used for pretreatment and upon the paraoxon dosage (Table 1). Ninety-sex percent of the animals that had received 3 µmol paraoxon died after 30 min. In contrast, only 13% of rats pretreated with K-27 before the same paraoxon exposure died after this time period (Table 1, first column). After 48 h, the mortality of rats that had only been given 3 µmol paraoxon but no pretreatment was 96%, whereas pretreatment with K-27 reduced the mortality to 42% after 48 h (Table 1, last column).

In contrast, the mortality rate of control rats that had only received equitoxic doses of the pretreatment compounds (pyridostigmine, physostigmine, ranitidine, tacrine, or K-27) but no paraoxon was 0%; i.e., all rats survived. Animal behavior was not systematically assessed, but no gross behavioral disturbances, i.e., salivation, lacrimation, twitches, or uncoordinated movements, were observed in these control animals. Some of the animals given physostigmine had loose bowel motions.

### 2.2. Survival Analysis

**Figure 2:**
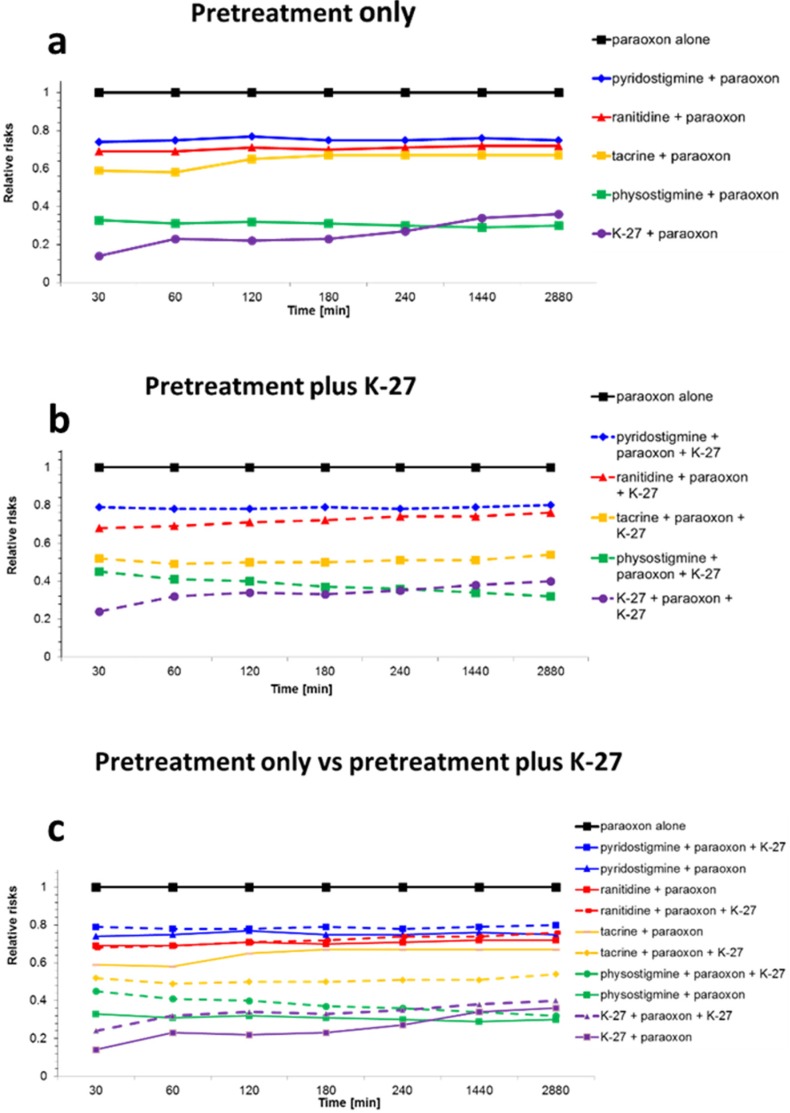
Relative risk (RR) of death estimated by Cox analysis [55] in animals that received prophylactically various cholinesterase inhibitors (pyridostigmine, physostigmine, ranitidine, tacrine, or K-27) 30 min before intraperitoneal (i.p.) paraoxon injections and either no additional treatment (solid lines) or additional K-27 injections (60 μmol/rat diluted in 500 μL saline solution; i.p.) (dashed lines). (**a**): Comparison of different pretreatment regimens without subsequent posttreatment. (**b**): Comparison of different pretreatment regimens followed by subsequent posttreatment with K-27. (**c**): Comparison between pretreatment without subsequent posttreatment (solid lines) and pretreatment followed by posttreatment with K-27 (dashed lines). The RR has been adjusted for paraoxon dose (high/low) at each of the time points examined (30 min, 1, 2, 3, 4, 24, and 48 h). The protective effect of different cholinesterase inhibitors, administered at a dosage of about one-fourth of the LD_01_, is compared to no pretreatment (black line: paraoxon alone, RR = 1). K-27 and physostigmine conferred best protection, followed by tacrine, ranitidine, and pyridostigmine. The difference between pretreatment before paraoxon exposure alone (pretreatment only, solid lines) and pretreatment plus K-27 posttreatment (dashed lines) was not statistically significant for any of the pretreatment regimens.

Figure 2a–c show the relative risk (RR) of death at the seven time points (30 min, 1, 2, 3, 4, 24, and 48 h) estimated by the Cox analysis in pretreated animals. It was compared with animals that had received paraoxon alone, but neither pre- nor posttreatment (group 1: RR = 1) and was adjusted for paraoxon dose (high/low). Statistical comparison (Table 2) between the different pre- and posttreatment regimens was performed on the cumulative relative risk, i.e., the area under the RR time curve (Figure 2).

Based on these data, pretreatment with all the tested compounds significantly (*p* ≤ 0.05) reduced the RR of animals exposed to paraoxon. Best protection from paraoxon-induced mortality was observed after pretreatment with physostigmine, reducing the risk to 30% (RR = 0.30), and K-27, reducing the risk to 34% (RR = 0.34). Both substances were significantly (*p* ≤ 0.05) more efficacious than the three other compounds tested for pretreatment: prophylactic administration of tacrine (RR = 0.67), ranitidine (RR = 0.72), and pyridostigmine (RR = 0.76) were less efficacious but still significantly (*p* ≤ 0.05) reduced the relative risk of death compared to the no-treatment group (G1: paraoxon only). When the pretreatment was combined with a subsequent K-27 posttreatment, all tested compounds, except pyridostigmine (RR = 0.91), significantly reduced paraoxon-induced mortality. Best protection was again observed for physostigmine (RR = 0.30) and K-27 (RR = 0.37), followed by tacrine (RR = 0.67) and ranitidine (RR = 0.77).

Additional administration of K-27 immediately after paraoxon exposure (posttreatment) did not further reduce mortality (Figure 2c), compared to pretreatment alone. Statistical analysis between pretreatment before paraoxon exposure alone (pretreatment only) and pretreatment plus K-27 posttreatment did not show any significant difference for any of the pretreatment regimens (Table 2).

## 3. Discussion

The purpose of this study was to determine if posttreatment with the experimental oxime K-27 reduces the mortality of rats pretreated with a group of known non-OPC AChE inhibitors and exposed to paraoxon thereafter. When comparing the efficacy of various compounds, they have to be administered in equivalent dosages. We decided to equidose according to in vivo toxicity, i.e., 25% of LD_01_, which is a quantity well-tolerated by the experimental animals [33]. Previously, we have discussed in detail why choosing dosages based on in vitro parameters—for instance, the IC_50_ for AChE inhibition—disregards toxicities unrelated to AChE inhibition and may yield false negative results [26,31,33].

Our results on animals pretreated with diverse cholinesterase inhibitors and exposed to the OPC paraoxon confirm the data obtained previously [26,31,47], demonstrating that best protection from OPC-induced mortality is obtained by physostigmine and K-27, which is superior to the protection afforded by pyridostigmine, the only FDA-approved prophylaxis when soman exposure is imminent [30]. Whereas physostigmine readily passes the blood-brain barrier, precluding its use in high-performance operational populations (reviewed by [5,26]), only a negligible proportion of K-27 enters the brain [56,57] making it a promising alternative to pyridostigmine when passage into the brain is not desired [26,47].

In addition, our data indicate that K-27 administrations immediately after paraoxon exposure of animals pretreated by the reversible OPC inhibitors physostigmine, pyridostigmine, ranitidine, tacrine, or K-27 does not further reduce mortality. This result was somewhat unexpected, since it is generally assumed that posttreatment with oximes improves the outcome of animals pretreated and exposed to OPCs thereafter [53]. Several explanations are conceivable: Inns and Leadbeater [52] could demonstrate that the efficacy of posttreatments in pretreated animals depends on the OPC and the dosage of the oxime used for posttreatment. They performed a systematic study pretreating guinea pigs with pyridostigmine, exposing them to the nerve agents sarin, soman, and VX and treating them thereafter with either trimedoxime or obidoxime in combination with atropine and diazepam. Pretreatment with pyridostigmine significantly improved the survival of animals exposed to all three OPCs. The protected ratio did not further improve by oxime posttreatment in animals exposed to soman, whereas a significant increase in the protective ratio was observed for both trimedoxime and obidoxime treatments in guinea pigs exposed to sarin and VX. This is, however, most probably related to the fact that trimedoxime and obidoxime are poor reactivators of soman-inhibited AChE [58]. In contrast, we have previously been able to show that K-27 very efficaciously protects from the lethal effects of paraoxon, reducing mortality in rats by 80% if administered after OPC exposure without pretreatment [45]. When comparing efficacies of oximes as pre- and posttreatment, it also needs to be considered that there are significant species differences in activities and the distribution of cholinesterases and in oxime efficacies between rats, mice, guinea pigs, and humans.

Another explanation may be the choice of dosage. Studies by Kassa and Fusek [59,60] indicate that oxime posttreatment is only efficacious if the OPC is applied in a very high, supralethal dosage. They performed two experiments: in the first study, they pretreated rats with pyridostigmine/benactyzine/trihexyphenidyle (“Panpal”) and exposed them to sublethal doses of soman [59]. In this case, subsequent oxime (HI-6) treatment did not improve the efficacy. In contrast, when the animals were exposed to a supralethal OPC dose, they only survived if the Panpal pretreatment was combined with an oxime posttreatment [60]. In our study, the experimental animals were exposed to paraoxon in the dosage of LD_70_–LD_95_, a dosage that may be too low to allow for postexposure K-27 treatment to be efficacious. It remains to be determined if the outcome of pretreatment is improved by K-27 posttreatment in animals exposed to much higher OPC dosages. 

When interpreting these data, it needs to be taken into account that K-27, when administered after paraoxon exposure without pretreatment, very efficaciously reduces paraoxon-induced mortality [45]. In practical terms, this indicates that pretreatment does not further improve the therapeutic outcome in exposure to sublethal OPC dosages if posttreatment facilities are available.

## 4. Materials and Methods 

### 4.1. Chemicals

Paraoxon stock solution (100 mmol/L) was prepared in dry acetone. Working solution for intraperitoneal (i.p.) application was prepared ex tempore by diluting the stock solution with saline shortly before application. Paraoxon (Paraoxon-ethyl PESTANAL^®^, product number: 36186, analytical standard), pyridostigmine (Pyridostigmine bromide, product number: P9797, purity [HPLC] ≥ 98%), physostigmine (Eserine, product number: E8375, purity [HPLC] ≥ 99%), ranitidine hydrochloride (product number: R101, purity [TLC] ≥ 98%), and tacrine (product number: A3773, purity [titration] ≥ 99%) were purchased from Sigma-Aldrich Chemie (Sigma-Aldrich Chemie GmbH, Steinheim, Germany). K-27 was synthesized in the Department of Toxicology at the Faculty of Military Health Sciences (University of Defense), Hradec Kralove, Czech Republic, according to Kuča et al. [37] and tested for purity by thin-layer chromatography (TLC) (Merck, Darmstadt, Germany) and high-performance liquid chromatography (HPLC) (Spectra-Physics Analytical, Fremont, CA, USA) described in detail by Jun et al. [61,62]. The water was distilled and deionized.

### 4.2. Experimental Animals

During the entire experiment, the “Guiding principles in the Care of and Use of Laboratory Animals” (Council of The American Physiological Society) have been observed. All studies were performed with the approval of the Institutional Review Board (CMHS Animal Research Ethics Committee, UAEU, approval No. A18/09).

The original stock of Wistar rats was purchased from Harlan Laboratories (Harlan Laboratories, Oxon, England). The animals used in the present studies were bred at our own Animal Facility from the original stock. Adult male rats (average weight ± SD: 248 ± 13 g; 95% confidence interval: 247–249 g) were housed in polypropylene cages (43 × 22.5 × 20.5 cm^3^; six rats/cage) in climate- and access-controlled rooms (23 ± 1 °C; 50% ± 4% humidity). The day/night cycle was 12 h/12 h. Food and water were available ad libitum. The food was standard maintenance diet for rats purchased from Emirates Feed Factory (Abu Dhabi, UAE).

#### 4.2.1. Choice of Dosage for Pretreatment

25% of LD_01_ [33] was considered a quantity well-tolerated by the experimental animals, and therefore, the following dosages were administered for pretreatment (Table 3):

- 2 (pre- and posttreatment) reference groups: only paraoxon exposure.
- Pyridostigmine: 1 µmol/rat = 0.26 mg/rat (= 1 mg/kg average body weight).
- Physostigmine: 0.25 µmol/rat = 0.07 mg/rat (= 0.28 mg/kg average body weight).
- Ranitidine hydrochloride: 12 µmol/rat = 4.21 mg/rat (= 17.0 mg/kg average body weight).
- Tacrine: 4 µmol/rat = 1 mg/rat (= 4.0 mg/kg average body weight).
- K-27: 60 µmol/rat = 26.77 mg/rat (= 108.0 mg/kg average body weight).

#### 4.2.2. Pretreatment, Paraoxon, and Oxime Exposure

In the experimental groups, animals received i.p. injections of paraoxon, in a dosage of either 1 µmol = 272 µg (1.1 mg/kg average body weight ≈LD_75_), 2 µmol = 544 µg (2.19 mg/kg average body weight), or 3 µmol = 816 µg (3.29 mg/kg average body weight) diluted in 500 µL saline solution. For each dosage, there were 11 groups of rats; the experiments were repeated four times (4 cycles; 6 rats/cycle). The first group (paraoxon) was given paraoxon i.p. alone. Groups 2–6 first received i.p. injections of the AChE inhibitor (pyridostigmine, physostigmine, ranitidine hydrochloride, tacrine, or K-27 diluted in 500 µL saline solution) and, 30 min later, a paraoxon injection. Groups 7–11 received the same pretreatment (i.p. injections of the AChE inhibitors (pyridostigmine, physostigmine, ranitidine hydrochloride, tacrine, or K-27 diluted in 500 µL saline solution); 30 min later, a paraoxon injection and, immediately thereafter (within one minute), an additional i.p. injection of the oxime-type reactivator K-27 (60 μmol/rat, diluted in 500 µL saline solution). The AChE inhibitors, the organophosphorus compound, and the oxime were injected at anatomically distinct sites, thereby minimizing the risk of interaction between the OPC, the AChE inhibitor, and the oxime in the peritoneal cavity.

The animals were monitored for 48 h, and mortality was recorded at 30 min, 1, 2, 3, 4, 24, and 48 h. There were 5 control groups, consisting of 6 rats each, which received only the prophylactic agent but no paraoxon injections.

### 4.3. Statistical Analysis

Statistical analysis was performed on the mortality data of four cycles. Mortality data were compared and, for each of the seven time points, the respective hazards ratios (relative risks of death) were estimated using the Cox proportional hazards model [55]. Both paraoxon doses (2 and 3 µmol/rat, respectively, with 1 µmol as the reference category) and groups, i.e., type of pre-/posttreatment (with group 1, i.e., no pretreatment, as the reference category), were treated as categorical variables.

Subsequently, the area under the RR time curve was determined, and pair-wise comparisons (Mann-Whitney U-Test) were performed in order to determine the most protective reactivator. No Bonferroni correction for multiple comparisons was applied, and an α ≤ 0.05 was considered significant. The SPSS 21.0 (IBM Corp. Armonk, NY, USA) software package was used for all statistical evaluations.

## 5. Conclusions

When administering physostigmine, pyridostigmine, ranitidine, tacrine, or K-27 at a dosage of 25% of LD_01_ as a pretreatment, best outcome is achieved if physostigmine or K-27 is administered prophylactically 30 min before exposure to paraoxon at a dosage between LD_70_ and LD_95_. The therapeutic outcome is not further improved by additional therapy with the experimental oxime K-27 immediately thereafter. This indicates that pretreatment may not yield an additional benefit in exposure to sublethal OPC dosages if posttreatment facilities are available.

## Figures and Tables

**Table 1:** Mortality of rats given paraoxon intraperitoneally (i.p.) in a dosage of 1 (first value), 2 (second value), or 3 µmol/animal (third value). Listed is the proportion of dead animals in percent (derived from 24 rats) at each time point (30 min, 1 h, 2 h, 3 h, 4 h, 24 h, and 48 h after paraoxon injection) for rats given no pretreatment (rows 1 and 2: paraoxon only) and for animals given i.p. injections of the AChE inhibitors pyridostigmine, physostigmine, ranitidine, tacrine, or K-27 30 min before paraoxon exposure (pretreatment), either alone or followed by K-27 immediately after paraoxon exposure (K-27 posttreatment). The dose injected for pretreatment and for K-27 posttreatment was approximately one-fourth of the LD_01_. The lines are arranged to compare pretreatment alone (white row) are listed above the same treatment combined with K-27 posttreatment (grey row, underneath).

Groups (G)	30 min	1 h	2 h	3 h	4 h	24 h	48 h
**Paraoxon only (pretreatment group)**	63/88/96	63/88/96	63/88/96	67/88/96	67/88/96	67/88/96	67/88/96
**Paraoxon only (pre- and posttreatment group)**	75/88/92	79/88/92	79/88/92	79/92/92	83/92/92	83/92/96	83/92/96
**Pyridostigmine pretreatment**	13/88/83	21/88/83	25/88/88	25/88/88	25/88/88	29/88/88	29/88/88
**Pyridostigmine pretreatment + K-27 posttreatment**	13/100/96	13/100/100	13/100/100	21/100/100	21/100/100	29/100/100	29/100/100
**Physostigmine pretreatment**	4/29/46	4/29/46	13/33/46	13/33/46	13/33/46	13/33/46	13/33/50
**Physostigmine pretreatment + K-27 posttreatment**	8/42/54	8/42/54	8/42/54	8/42/54	8/42/54	8/42/54	8/42/54
**Ranitidine pretreatment**	0/79/92	0/83/92	4/92/ 92	4/92/92	8/92/92	13/92/92	13/92/92
**Ranitidine pretreatment + K-27 posttreatment**	25/58/83	29/67/83	33/67/83	38/75/83	42/83/83	46/83/83	46/96/83
**Tacrine pretreatment**	0/54/92	0/58/92	13/71/92	13/79/92	13/79/92	13/79/92	13/79/92
**Tacrine pretreatment + K-27 posttreatment**	0/63/83	0/63/83	0/67/83	0/71/88	0/71/92	0/75/92	0/79/92
**K-27 pretreatment**	13/8/13	25/17/21	25/21/25	25/21/29	33/29/33	46/42/38	46/42/42
**K-27 pretreatment + K-27 posttreatment**	0/25/33	17/25/46	21/25/46	21/29/46	21/42/46	25/54/50	25/58/50

**Table 2:** Cox analysis of the cumulative relative risk (RR) of death, including 95% confidence interval (CI), of animals injected with paraoxon intraperitoneally (i.p.) and adjusted for paraoxon dose (high/low). The cumulative RR was assessed by determining the area under the RR time curve (see Figure 2) for pre-exposure treatment with the AChE-inhibitors pyridostigmine, physostigmine, ranitidine, tacrine, and K-27 (pretreatment), either alone or followed by K-27 immediately after paraoxon exposure (K-27 posttreatment). The injected dose was approximately one-fourth of the LD_01_. Group 1, i.e., paraoxon only and no pretreatment, was the reference category (RR = 1). Listed are mean values ± standard deviations (SD). Statistical differences compared to the reference group (only paraoxon and no pretreatment) were tested by the Mann-Whitney U-Test and a *p*-value ≤ 0.05 was considered significant. Best protection was observed for K-27 and physostigmine, reducing the cumulative mortality to about 30% (RR ≈ 0.30–0.34), which is significantly better than the three other tested compounds. Tacrine, ranitidine, and pyridostigmine were less efficacious (RR ≈ 0.67–0.76) but still significantly reduced the relative risk of death. The differences between pretreatment before paraoxon exposure alone (pretreatment only) and pretreatment plus K-27 posttreatment were not statistically significant for any of the pretreatment regimens.

Groups	Relative Risk (RR)	95% CI	*p*-Value
Paraoxon-ethyl only	1	1 --- 1	---
Pyridostigmine + paraoxon-ethyl	0.76 ± 0.13	0.54–0.97	≤0.05 ^a^
Pyridostigmine + paraoxon-ethyl+K-27	0.91 ± 0.14	0.69–1.13	≤0.05
Physostigmine + paraoxon-ethyl	0.30 ± 0.15	0.06–0.53	≤0.05 ^a,b^
Physostigmine + paraoxon-ethyl+K-27	0.32 ± 0.13	0.11–0.54	≤0.05 ^a^
Ranitidine + paraoxon-ethyl	0.72 ± 0.16	0.46–0.98	≤0.05 ^a^
Ranitidine + paraoxon-ethyl+K-27	0.77 ± 0.10	0.61–0.93	≤0.05 ^a^
Tacrine + paraoxon-ethyl	0.67 ± 0.21	0.33–1.00	≤0.05 ^a^
Tacrine + paraoxon-ethyl+K-27	0.59 ± 0.12	0.41–0.78	≤0.05 ^a^
K-27+ paraoxon-ethyl	0.34 ± 0.09	0.20–0.48	≤0.05 ^a,b^
K-27+ paraoxon-ethyl+K-27	0.37 ± 0.08	0.24–0.49	≤0.05 ^a^

^a^*p* ≤ 0.05 compared to paraoxon-ethyl alone (no pretreatment). ^b^ *p* ≤ 0.05 compared to pyridostigmine, ranitidine, and tacrine pretreatment.

**Table 3:** Chemical and biological parameters of the acetylcholinesterase (AChE) inhibitors tested prophylactically before paraoxon exposure. Column 2 lists their molecular weight, column 3 their concentration necessary to inhibit 50% of human red blood cell AChE activity (IC_50_), column 4 their LD_50_ and LD_01_ values for intraperitoneal (i.p.) application in rats [33], and columns 5–7 the doses injected i.p. for pretreatment before paraoxon exposure. Values are given in µmol/animal (column five), mg/animal (column six), and in mg/kg average body weight (column seven). The injected dose is approximately one-fourth the LD_01_.

	Molecular Weight	IC_50_ * [µM]	LD_50_/LD_01_ * (µmol/rat)	Injected Dose (≈ ¼ of LD_01_) (µmol/rat)	Injected Dose (mg/rat)	Injected Dose (mg/kg Average Body Weight )
Pyridostigmine	261.12	0.330	7.2/3.7	1	0.26	1
Physostigmine (Eserine)	275.35	0.012	3.0/0.9	0.25	0.07	0.28
Ranitidine	350.86	2.5	59/46	12	4.21	17.0
Tacrine	250	0.200	21.5/16	4	1	4.0
K-27	446.16	414	350/250	60	26.77	108.0

* data from [33].
